# The Influence of Ionizing Radiation, Temperature, and Light on Eplerenone in the Solid State

**DOI:** 10.1155/2014/571376

**Published:** 2014-08-04

**Authors:** Katarzyna Dettlaff, Magdalena Ogrodowczyk, Witold Kycler, Agnieszka Dołhań, Barbara Ćwiertnia, Piotr Garbacki, Anna Jelińska

**Affiliations:** ^1^Department of Pharmaceutical Chemistry, Poznan University of Medical Sciences, Grunwaldzka 6, 60-780 Poznań, Poland; ^2^Department of Oncological Surgery II, Greater Poland Cancer Centre, Garbary 15, 61-866 Poznań, Poland; ^3^Department of Inorganic and Analytical Chemistry, . Poznan University of Medical Sciences, Grunwaldzka 6, 60-780 Poznań, Poland

## Abstract

Eplerenone was subjected to the influence of ionizing radiation in the form of a high-energy electron beam (25–400 kGy), high temperature (90°C RH 0% and 60°C RH 76.4%), and light (6 mln lux h). An HPLC method was used to determine the content of eplerenone and to establish the impurity profile of all samples. As eplerenone was found to be a compound of great resistance to the above stress factors with the exception of high doses of ionizing radiation (≥200 kGy) when its degradation was above 1%, it is possible to sterilize eplerenone by radiation method with the standard dose of 25 kGy. Based on the analysis of impurities and degradation products, the mechanism of radiodegradation was demonstrated to differ from the mechanisms of photo- and thermodegradation. The observation that the DSC curves for the nondegraded and degraded samples of eplerenone were significantly different only under exposure to the electron beam confirmed the applicability of DSC for studies of radiolytic degradation of eplerenone.

## 1. Introduction

**Figure 1:**
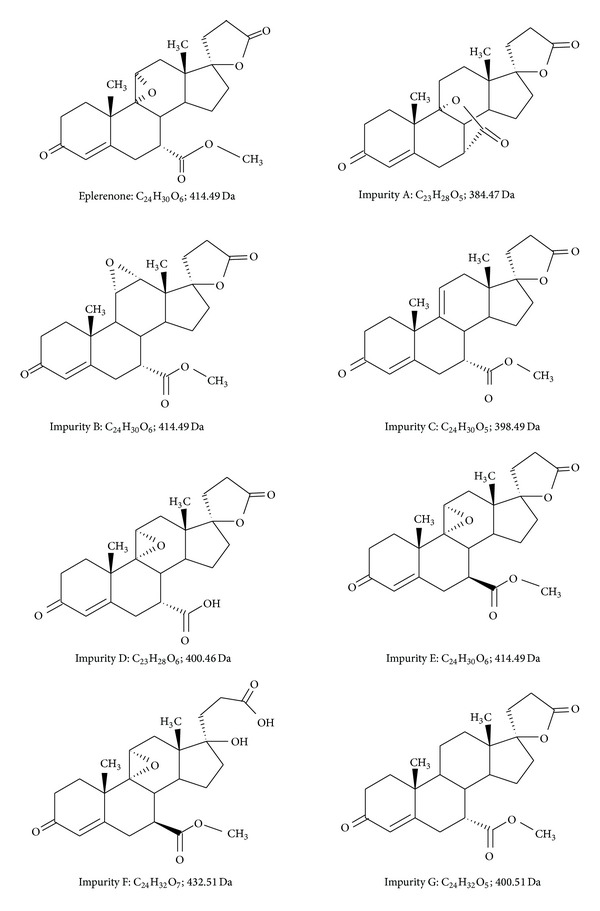
Structural formulas of eplerenone and its potential impurities [20].

Eplerenone (pregn-4-ene-7,21-dicarboxylic acid, 9,11-epoxy-17-hydroxy-3-oxo, *γ*-lactone, methyl ester (7*α*, 11*α*, 17*α*)—IUPAC—Figure 1) is an aldosterone antagonist used in the treatment of hypertension and cardiac insufficiency [1].

What distinguishes it from spironolactone, the first aldosterone antagonist in use for over 50 years, is the epoxy bridge between positions 9*α* and 11*α* in the cyclopentanoperhydrophenanthrene ring system and the methoxycarboxyl group at position 7*α* [2]. Due to those chemical modifications eplerenone exhibits desired selectivity for the mineralocorticoid receptor. The affinity of eplerenone for androgen and progesterone receptors is 100 times less in comparison to spironolactone, which reduces the risk of unwanted effects such as gynecomastia, impotence, or menstruation disorders [1–3].

*In vitro *and* in vivo* studies explained the metabolism of eplerenone [4, 5] as induced by cytochrome P450 enzyme CYP3A4, with the main metabolites 6*β*-hydroxyeplerenone and 21-hydroxyeplerenone.

Previous studies of the stability of eplerenone [6–9] indicated its considerable resistance to light and high temperature in the solid state and susceptibility to acid and base degradation in solution. Rane et al. observed a loss of eplerenone in an oxidizing environment (30% H_2_O_2_, 60°C, 6 h), but the study did not involve identification of degradation products [6]. Sudhakar Babu et al. by using an LC-MS method identified methyl hydrogen 9,11,17-trihydroxy-3-oxopregn-7-*α*-carbonate, 21*α*-carboxylic acid as an eplerenone degradation product occurring under exposure to 0.5 mol/L NaOH [7]. Sonawane and Gide [8, 9] used 2 or 3 different concentrations of acids, bases, and H_2_O_2_ as well as various values of temperature, relative air humidity, and time in order to apply the multiple regression equation for data analysis. Yate's algorithm showed that eplerenone was the most vulnerable to the influence of NaOH and depended on exposure time, whereas the effect of temperature was less significant. By using greater concentrations of degrading factors than those applied by Sudhakar Babu et al., it was possible to identify more degradation products (Table 2).

The radiochemical stability of eplerenone has not been studied so far. Sterilization and decontamination of drugs by using gamma or e-beam ionizing irradiation has been recommended by the European Pharmacopoeia (Ph. Eur.) 7th Edition [10] for a long time and is becoming increasingly common [11, 12].

Although it is estimated that approximately 90% of medicinal substances in the solid state may be sterilized in that manner, it is necessary to determine whether the standard dose of 25 kGy does not damage the drug structure [11]. It is also possible to use a lower dose of irradiation after demonstrating that it ensures effective sterilization (dose validation). The efficacy of irradiation sterilization is affected by three basic parameters: level of initial microbial impurity, sterility assurance level (SAL), and microorganism irradiation sensitivity [13].

Research into drug irradiation stability is increasingly applying high doses of ionizing radiation (100–800 kGy) [14, 15], which can be compared to stress studies that allow establishing any physicochemical changes in a substance tested as well as permitting development and validation methods for determination of radiodegradation products.

The resistance of steroid drugs to ionizing radiation has been investigated since the 1980s [11, 16–19] with a focus on anti-inflammatory natural and synthetic glucocorticosteroids. In addition to chromatographic methods, the use of differential scanning calorimetry (DSC) in resistance studies is becoming more widespread [19].

The purpose of this work was to investigate the radiochemical stability of eplerenone in order to determine whether it may be sterilized by means of irradiation. The use of doses exceeding 25 kGy (50–400 kGy) was designed to identify eplerenone degradation products and to compare them with those reported in the literature. The study involved the application of DSC to evaluate samples of eplerenone during radio-, thermo-, and photodegradation, with the aim of verifying the suitability of that method for a study of eplerenone stability.

## 2. Material

Eplerenone and its impurities were obtained from Industriale Chimica s.r.l., Saronno, Italy. The structural formulas of impurities A–G are shown in Figure 1.

All other chemicals and solvents were obtained from Merc KGaA (Germany) and were of analytical grade. High quality pure water was prepared using a Millipore purification system (model Exil SA 67120, Millipore, Molsheim, France).

## 3. Methods

### 3.1. Irradiation with E-Beam Radiation

Approximately 0.5 g of eplerenone was placed in 4 mL colourless glass jars closed with a plastic stopper and irradiated to 25, 50, 100, 200, and 400 kGy with the e-beam from a linear electron accelerator Elektronika 10/10. The energy of electrons was 9.96 MeV and the current intensity 6.2 *μ*A.

### 3.2. Photodegradation Experiments

Approximately 10 mg of eplerenone was placed in 4 mL colourless vials and illuminated with a SUNTEST CPS+ device (Heraeus, Germany). In the photodegradation studies that were consistent with the ICH Q1B guidelines the following conditions were applied: a 1500 W lamp, a 300–800 nm wavelength range, an ID65 solar filter, and an irradiation intensity of 250 Wm^−2^. Exposure times of 21.6 and 108 hours provided an overall illumination of not less than 1.2 million and 6 million lux hours, respectively. A 10 mg control sample of eplerenone in a glass vial was wrapped in aluminium foil.

### 3.3. Thermodegradation Experiments

10 mg samples of eplerenone were placed in 4 mL vials and put in heat chambers at 90°C (RH 0%) and 60°C (RH 76.4%). At specified time intervals (1, 3, 5, and 7 days), determined by the rate of degradation, the vials were removed and cooled to room temperature.

### 3.4. High Performance Liquid Chromatography [20]

The analytical system (consisted of a quaternary pump L-7100, an autosampler L-7200, a column oven L-7360, and a diode array detector L-7455; all are Merck Hitachi products) was used for chromatographic separation of the impurities and degradation products of eplerenone samples. All the samples (2.5 mg/mL) were dissolved in the solvent mixture (methanol, acetonitrile, and water 25 : 25 : 50 V/V/V). An Inertsil ODS3 C18 (150 × 4.6 mm, 3 *μ*m) analytical column was employed as a stationary phase; the column temperature was 30°C. The mobile phase consisted of solution A (0.1% phosphoric acid in water) and solution B (methanol, acetonitrile, and phosphoric acid 60 : 40 : 0.1 V/V/V).

The gradient system was as in Table 1.

UV detection was performed at 240 nm. The flow rate was 1.0 mL/min and the injection volume 20 *μ*L.

### 3.5. Differential Scanning Calorimetry

Measurements were performed with DSC-50 Shimadzu, Japan. 2 mg samples were sealed in aluminium crucibles with pierced lids. The samples were thermally equilibrated at 20°C for 5 min and the measurements were performed at a heating rate of 5°C min^−1^ in a nitrogen atmosphere (30 mL min^−1^). For each sample, three independent measurements were performed and the results were averaged.

## 4. Results and Discussion

**Figure 2:**
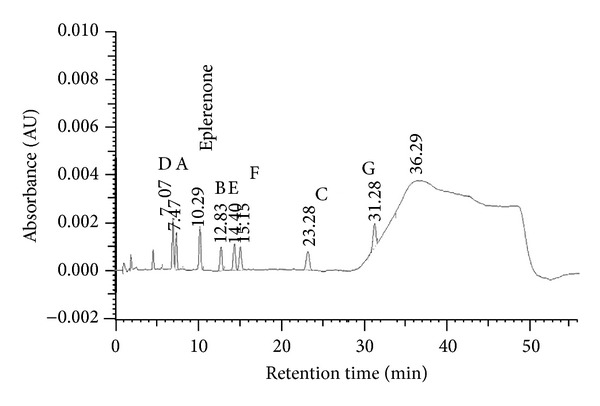
Chromatograms of eplerenone and its 7 potential impurities.

The eplerenone samples subjected to the influence of temperature (in dry air and at 76.4% RH), light, and ionizing radiation and the control sample (not exposed to the stress conditions) were analyzed chromatographically. The HPLC method, previously described [20], was revalidated taking into consideration selectivity. Chromatograms of eplerenone and its 7 potential impurities (at a concentration of 0.1% each) were obtained (Figure 2) and a relative response factor (RRF) for all potential impurities was calculated.

**Figure 3:**
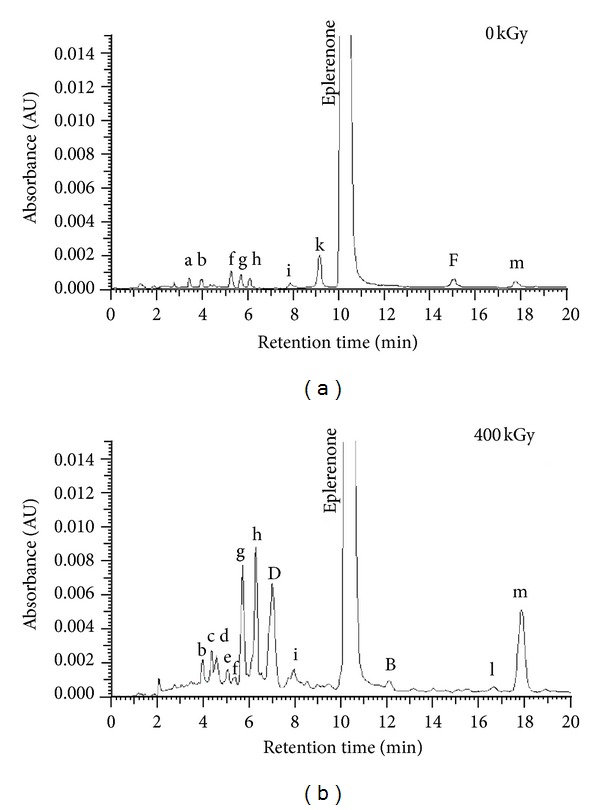
Chromatograms of eplerenone before (a) and after irradiation (b).

Based on the chromatograms of the degraded and nondegraded samples (Figure 3), the areas of peaks were found and the content of compounds of known chemical structure as well as the recovery of unknown compounds (RRF = 1) was calculated (Tables 3 and 4).

In the compounds of known chemical structure the presence of 4 impurities was detected: A, B, D, and F (identificated by comparison of retention times to standards). The nondegraded eplerenone sample was found to contain impurity F with a hydrolyzed lactone bond, whose small amount (0.022–0.051%) was also detected in the samples heated in dry air and those exposed to light. In the eplerenone samples exposed to light and heated trace amounts of impurity A (0.008%) were detected. Ionizing radiation proved to be the most destructive as only in the irradiated samples impurities D (≥25 kGy)—a compound with a hydrolyzed ester bond—and B (≥100 kGy)—an eplerenone isomer—were found.

14 compounds of unknown structure were found, of which 9 were present in the control sample that contained 99.78% eplerenone. After storage at 60°C (RH 76.4%) and 90°C (RH 0%) over a period of 1 week, the eplerenone content was slightly lower, 99.74% and 99.60%, respectively. Exposure to light for 110 h in the SUNTEST CPS+ chamber, simulating the energetic composition of sunlight, was equivalent to a dose of 6 mln lux h, which was 5 times greater than the minimum value recommended for photostability studies. According to the ICH guidelines [21], if a medicinal substance manifests its degradation after receiving a 1.2 lux h dose, it is considered photolabile. When its content and physicochemical parameters are acceptable following exposure to 6 mln lux h, the substance is referred to as photostable. Eplerenone may be regarded as photostable because its loss on exposure to light was 0.13% relative to the control sample.

**Figure 4:**
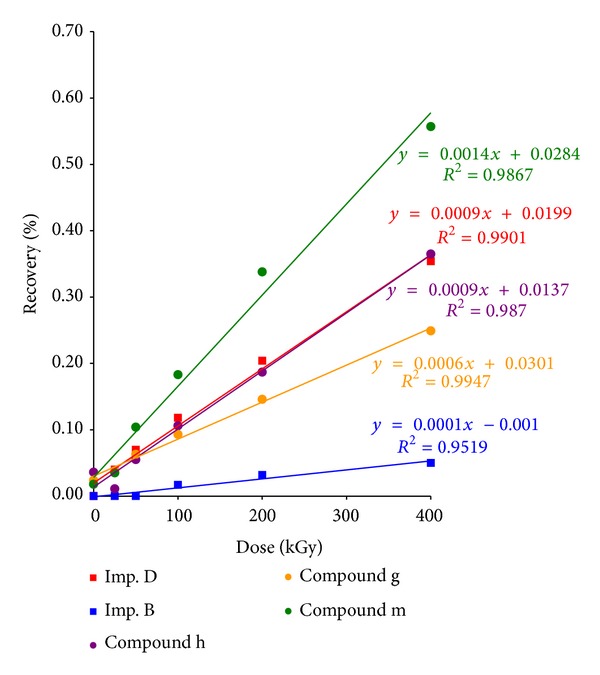
Dependence of eplerenone radiodegradation product recovery on radiation dose.

Although the standard dose of sterilization irradiation also did not have a destructive effect on eplerenone, it was found that the highest dose (400 kGy) resulted a loss of 1.78%. It was also observed that different degradation products were formed than during termo- and photodegradation. For impurities B and D as well as the compounds of unknown structure, a relationship was identified between the content or recovery and the dose of ionizing radiation (Figure 4).

The next stage of this work was to analyze the nondegraded samples of eplerenone and those affected by stress factors with the use of differential scanning calorimetry. The DSC curve for the control sample displayed a typical endothermic melting peak with a maximum at 248.8°C. After the thermo- and photodegradation of eplerenone, the DSC curves had similar melting peak parameters (Table 5).

**Figure 5:**
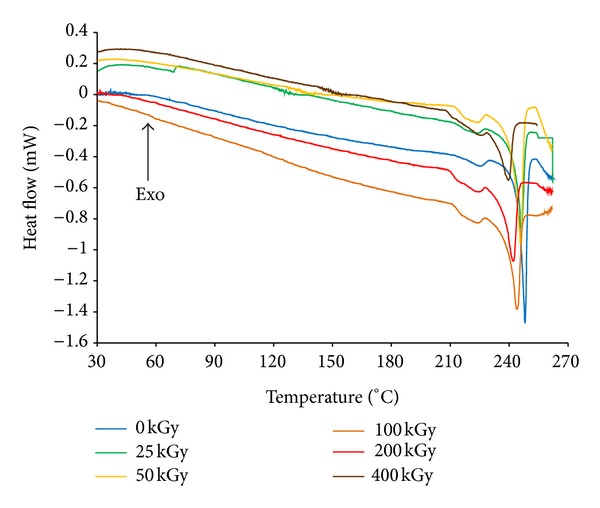
The DSC curves of degraded and nondegraded eplerenone.

The DSC curves for radiodegradation varied distinctly from that for the control sample (Figure 5). The melting peak moved towards lower values and at 25 kGy this difference was 2.4°C, while at 400 kGy, it was 8.9°C. The parameters *T*_endset_ for the control sample and for those irradiated shifted similarly to the parameters *T*_max⁡_. Small irregularities in the curve courses were observed regarding *T*_onset_ (Table 5) and resulted from the difficulty in determining the temperature of the melting peak onset as the melting peak was preceded by a slight endothermic effect, probably due to the presence of polymorphic forms of eplerenone. However, the quantitative ratio of forms H to L did not change after irradiation.

## 5. Conclusion

Eplerenone is a compound exhibiting a high resistance to temperature, light, and ionizing radiation. That makes it suitable for irradiation sterilization and decontamination as a dose of 25 kGy caused merely a 0.13% loss of content, of which 0.04% was found to be impurity D—a derivative with a hydrolyzed ester bond. Its content was observed to increase in proportion to the growth of ionizing radiation and reached 0.35% at 400 kGy.

The method based on differential scanning calorimetry proved useful for the evaluation of eplerenone radiodegradation since a degradation level of 1.78% (400 kGy) was seen in the DSC curve as a shift of the endothermal melting peak maximum towards lower values by as much as 8.9°C.

## Figures and Tables

**Table 1:** 

	Mobile phase A	Mobile phase B
0–25 min	54	46
25–32 min	54→40	46→60
32–45 min	40	60
45–46 min	40→54	60→46
46–56 min	54	46

**Table 2:** Degradation products of eplerenone [7, 8].

Stress conditions and duration	Degradation of eplerenone [%]	Molecular formula	Molecular weight [Da]	Reference
0.5 mol/L NaOH, 1 h, 25°C	20.3	C_24_H_34_O_8_	450.52	[7]

1 mol/lL NaOH, 2 h, 100°C	93.0	C_24_H_32_O_7_	432.51	[8]

1 mol/lL HCl, 2 h, 100°C	90.0	C_23_H_32_O_5_	388.50	[8]
		C_23_H_28_O_6_	400.46	[8]
		C_22_H_32_O_2_	328.49	[8]

**Table 3:** Eplerenone and impurities of known structure in degraded and nondegraded samples.

Conditions	Content [%]				
	Eplerenone (10.23 min)	Imp. D (7.07 min)	Imp. A (7.47 min)	Imp. B (12.83 min)	Imp. F (15.15 min)
Nondegraded	99.782	—	—	—	0.044
6 mln lux h	99.657	—	0.008	—	0.031
90°C RH 0%	99.598	—	0.007	—	0.022
60°C RH 76.4%	99.737	—	0.007	—	0.051
25 kGy	99.772	0.040	—	—	—
50 kGy	99.559	0.070	—	—	—
100 kGy	99.329	0.118	—	0.017	—
200 kGy	98.758	0.204	—	0.032	—
400 kGy	98.006	0.354	—	0.050	—

**Table 4:** Eplerenone and impurities of unknown structure in degraded and nondegraded samples.

Conditions	Recovery∗ [%]													
	a 3.57 min	b 3.79 min	c 4.40 min	d 4.64 min	e 5.15 min	f 5.49 min	g 5.87 min	h 6.48 min	i 8.03 min	j 8.83 min	k 9.17 min	l 16.64 min	m 17.89 min	n 19.71 min
Nondegraded	0.015	0.012				0.030	0.024	0.036	0.031		0.01		0.018	
6 mln lux h	0.013	0.014			0.112	0.033	0.027	0.035	0.021	0.038	0.009		0.021	
90°C RH 0%	0.010	0.014			0.009	0.034	0.028	0.035	0.139	0.017	0.011		0.023	
60°C RH 76.4%	0.012	0.013			0.009	0.070	0.025	0.035	0.017				0.025	
25 kGy	0.022	0.011					0.038	0.011	0.012			0.019	0.035	0.021
50 kGy		0.022	0.010				0.063	0.055	0.037			0.030	0.104	0.019
100 kGy		0.026	0.018	0.021			0.093	0.106	0.024			0.021	0.183	
200 kGy		0.034	0.033	0.037	0.022	0.017	0.146	0.187	0.056			0.025	0.338	
400 kGy		0.049	0.063	0.064	0.033	0.021	0.249	0.365	0.065			0.026	0.557	

*Content of unknown impurities calculated for their relative response factor equal to that of eplerenone.

**Table 5:** Results of DSC analysis. The values in parentheses are standard deviation.

Conditions	Temperature [°C]			Difference [°C]		
	*T*_onset_	*T*_max⁡_	*T*_endset_	*T*_onset_*E*__ − *T*_onset_	*T*_max⁡_*E*__ − *T*_max⁡_	*T*_endset_*E*__ − *T*_endset_
Nondegraded	234.5 (3)	248.5 (0)	253.5 (3)	—	—	—
6 mln lux h	232.6 (0)	248.3 (3)	253.2 (0)	−1.9	−0.2	0.0
90°C RH 0%	232.3 (6)	248.5 (0)	253.7 (3)	−2.2	0.0	−0.3
60°C RH 76.4%	231.7 (3)	248.2 (7)	253.6 (7)	−2.8	−0.2	+0.2
25 kGy	227.6 (7)	246.1 (3)	249.8 (3)	−6.8	−2.4	−3.7
50 kGy	227.3 (3)	245.7 (0)	249.3 (7)	−7.2	−2.8	−4.1
100 kGy	226.4 (3)	244.1 (3)	247.8 (3)	−8.1	−4.4	−5.7
200 kGy	226.7 (3)	242.0 (7)	246.5 (0)	−7.8	−6.4	−7.0
400 kGy	227.5 (0)	239.6 (3)	242.5 (7)	−7.0	−8.9	−10.9

*T*_onset_*E*__: melting peak onset for nondegraded eplerenone.

*T*_max⁡_*E*__: melting peak maximum for nondegraded eplerenone.

*T*_endset_*E*__: melting peak endset temperature for nondegraded eplerenone.
